# Biomarker MET in der Tumorpathologie

**DOI:** 10.1007/s00292-023-01189-2

**Published:** 2023-03-21

**Authors:** Michaela Angelika Ihle, Carina Heydt

**Affiliations:** Institut für Pathologie. Medizinische Fakultät und Uniklinik Köln, Universität zu Köln, 50937 Köln, Deutschland

**Figure 1:**
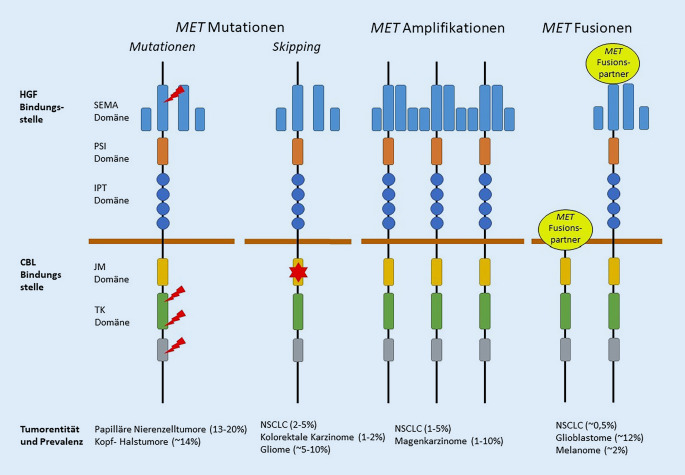

Das *MET*-Gen („MET proto-oncogene, receptor tyrosine kinase“), bestehend aus 21 Exonen, ist auf Chromosom 7q21-31 lokalisiert und codiert die MET-Rezeptortyrosinkinase (180 kDa), welche zusammen mit seinem Liganden, dem „hepatocyte growth factor“ (HGF) eine wichtige Rolle in der Tumorproliferation, Angiogenese und Migration spielt [1]. Eine Vielzahl von *MET*-Aberrationen, die zur Dysregulation des *MET*-Onkogens und somit zur Aktivierung verschiedener Signalwege wie MAPK, PI3K-AKT und JAK-STAT führen, wurden bereits beschrieben. Zu diesen zählen MET-Überexpression, *MET*-Genamplifikationen, *MET*-Mutationen einschließlich Exon-14-Skipping-Mutationen sowie *MET*-Fusionen (Abb. 1; [1]). Patienten mit einer solchen Aberration können zielgerichtet mit einem MET-Inhibitor behandelt werden [2]. Die hochselektiven und potenten MET-Inhibitoren Capmatinib (Tabrecta®, Novartis AG, Basel, Schweiz) und Tepotinib (Tepmetko®, Merck KGaA, Darmstadt, Deutschland) sind zugelassen für die Therapie von Patienten mit einem fortgeschrittenen, nichtkleinzelligen Lungenkarzinom (NSCLC) und *MET*-Exon-14-Skipping-Mutation, die eine systemische Therapie nach platinbasierter Chemotherapie und/oder einer Behandlung mit Immuntherapie benötigen. Weniger potente Multikinaseinhibitoren zur Behandlung von *MET*-Aberrationen wie Cabozantinib und Crizotinib befinden sich zurzeit in klinische Studien [2].

*MET*-Exon-14-Skipping-Mutationen treten in 3–4 % der NSCLC auf und sind sehr heterogen [3]. Bislang wurden über 100 verschiedene Mutationen beschrieben, die zu einem Skipping führen können [4]. Durch den Verlust der Juxtamembrandomäne und somit der Casitas-B-Lineage-Lymphoma(c-CBL)-Bindungsstelle erfolgt keine Degradierung von MET und der MET-Signalweg wird aktiviert. Neben *MET*-Exon-14-Skipping-Mutationen ist auch der Nachweis von Mutationen in Exon 16–19 der Kinasedomäne wichtig, da im NSCLC in diesem Bereich typische Resistenzmutationen gegenüber MET-Inhibitoren auftreten können. Auch im papillären Nierenzellkarzinom und anderen Karzinomarten wurden Mutationen in diesem Bereich nachgewiesen [3].

Therapien für NSCLC mit *MET*-Amplifikationen (3–5 %), die sowohl als primäre Treiberaberration als auch als Resistenzmechanismus gegenüber anderen Kinaseinhibitoren auftreten, befinden sich zurzeit in zahlreichen Studien. Hierbei zeigen sich MET-Inhibitoren besonders wirksam bei hochamplifizierten („high-level“) Tumoren [2]. Neben *MET*-Amplifikation befinden sich auch Tumoren mit *MET*-Fusionen in klinischen Studien. Die Frequenz der *MET*-Fusionen ist insbesondere in NSCLC allerdings sehr gering (< 0,5 %) und die Fusionspartner sind sehr heterogen, wie z. B. *TPR, CD74* und *KIF5B*. Ausnahme sind die Glioblastome, wo *MET*-Fusionen in 12 % der Fälle beschrieben sind (Abb. 1; [1, 3]).

Für den molekularpathologischen Nachweis der verschiedenen *MET*-Aberrationen ist die Implementierung hochwertiger und sensitiver Nachweisverfahren essenziell.

*MET*-Exon-14-Skipping-Mutationen können auf DNA- und RNA-Ebene detektiert werden. Hierbei werden vor allem amplikon- oder hybridisierungsbasierte Next-Generation-Sequencing(NGS)-Verfahren angewandt. Einzelgenanalysen wie Sanger-Sequenzierung oder RT-PCR können diese Aberrationen zwar nachweisen, werden aufgrund der Vielzahl von Biomarkern, die getestet werden müssen, und der geringen Materialverfügbarkeit immer seltener angewendet. Auf DNA-Ebene kann die Mutation nach der HGVS-Nomenklatur spezifiziert werden, allerding kann der Splicingeffekt nur auf RNA-Ebene nachgewiesen werden. Zudem haben Studien gezeigt, dass die Analyse mittels amplikonbasierter NGS-Verfahren auf DNA-Ebene zu falsch negativen Ergebnissen führen kann, wenn die Mutation an der Primer-Bindungsstelle auftritt [5].

Der Goldstandard für die Detektion von *MET*-Amplifikationen ist immer noch die Fluoreszenz-in-situ-Hybridisierung (FISH), die zurzeit DNA-basierten Verfahren immer noch überlegen ist. Zweifarbige FISH-Sonden markieren das *MET*-Gen und das Zentromer von Chromosom 7 (Cen7). Anhand von Auswertekriterien wie dem MET/CEN7-Verhältnis und/oder der durchschnittlichen *MET*-Genkopienzahl werden die Fälle in niedrig, intermediär und hochamplifiziert eingeteilt. Der Nachweis von *MET*-Amplifikationen bzw. Genkopienzahlveränderungen mittels DNA-basierten Verfahren befindet sich noch in der Evaluierung. Bisherige Studien haben gezeigt, dass ein geringer Tumorzellgehalt, Tumorheterogenität, fokale Amplifikationen, amplikonbasierte NGS-Verfahren und ctDNA anstelle von DNA ein Problem bei der Auswertung darstellen [6, 7].

Für den Nachweis von *MET*-Fusionen ist ein hybridisierungsbasiertes oder ein anchored-multiplex-PCR-basiertes NGS-Verfahren auf RNA-Ebene die erste Wahl [8]. Hiermit können sowohl unbekannte Fusionspartner als auch die involvierten Exons bestimmt werden. Alternativ können auch FISH-Analysen oder RT-PCR genutzt werden. Jedoch ist eine RNA-basierte NGS-Panelanalyse der sinnvollste Ansatz, da hier alle relevanten Fusionen sowie Exon-14-Skipping-Mutationen in nur einem Assay nachgewiesen werden können. DNA-basierte NGS-Ansätze, die sowohl Mutationen als auch Fusionen nachweisen können, haben sich in der Vergangenheit als wenig zuverlässig herausgestellt und führen oft zu falsch negativen Ergebnissen. Grund dafür ist, dass die Fusionsbruchpunkte oft große intronische Bereich umfassen und in repetitiven Regionen liegen, die schwer abzudecken sind [1].

Zusammenfassend zeigt die Vielzahl an *MET*-Aberrationen in NSCLC und anderen Karzinomen die wichtige Rolle dieser Kinase in der zielgerichteten Therapie und die Notwendigkeit einer flächendeckenden und qualitätsgesicherten molekularen Diagnostik.
